# Detecting Errors with Zero-Shot Learning

**DOI:** 10.3390/e24070936

**Published:** 2022-07-06

**Authors:** Xiaoyu Wu, Ning Wang

**Affiliations:** 1 School of Computer and Information Technology, Beijing Jiaotong University, Beijing 100044, China; 16112087@bjtu.edu.cn; 2 Beijing Key Laboratory of Traffic Data Analysis and Mining, Beijing 100044, China

**Keywords:** error detection, zero-shot learning, self-attention mechanism

## Abstract

Error detection is a critical step in data cleaning. Most traditional error detection methods are based on rules and external information with high cost, especially when dealing with large-scaled data. Recently, with the advances of deep learning, some researchers focus their attention on learning the semantic distribution of data for error detection; however, the low error rate in real datasets makes it hard to collect negative samples for training supervised deep learning models. Most of the existing deep-learning-based error detection algorithms solve the class imbalance problem by data augmentation. Due to the inadequate sampling of negative samples, the features learned by those methods may be biased. In this paper, we propose an AEGAN (Auto-Encoder Generative Adversarial Network)-based deep learning model named SAT-GAN (Self-Attention Generative Adversarial Network) to detect errors in relational datasets. Combining the self-attention mechanism with the pre-trained language model, our model can capture semantic features of the dataset, specifically the functional dependency between attributes, so that no rules or constraints are needed for SAT-GAN to identify inconsistent data. For the lack of negative samples, we propose to train our model via zero-shot learning. As a clean-data tailored model, SAT-GAN tries to recognize error data as outliers by learning the latent features of clean data. In our evaluation, SAT-GAN achieves an average $F_{1}$-score of 0.95 on five datasets, which yields at least 46.2% $F_{1}$-score improvement over rule-based methods and outperforms state-of-the-art deep learning approaches in the absence of rules and negative samples.

## 1. Introduction

Errors are very common in relational data, and just a small percentage [1] of errors in data cells will have terrible consequences for data analysis and data management. In general, we classify error data as inconsistent data, missing data, outdated data, and duplicate data. Error detection in relational tables that strive to find incorrect entries in a dataset is a critical step of data cleaning. Naturally, plenty of extensive research has been focused on it and different kinds of methods are proposed to detect errors, especially for inconsistent data. At present, error detection algorithms are typically categorized as rule-based methods, external information-based methods, and statistical learning-based methods.

Rule-based methods tend to solve the problem by judging whether the data violate the given rules (including integrity constraint and matching rules) or not. Generally, most error detection rules are predefined by experts, and rule making will have significant costs when the volume of data is very big. Despite yielding encouraging performance over many error detection tasks, rule-based approaches heavily depend on accurate and appropriate rules, which may become a bottleneck in data processing. The external information-based methods perform better than the rule-based methods as they take the knowledge bases or crowd-sourcing as master data. For example, the error detection algorithm based on a knowledge base identifies whether the input data match the data in the knowledge base to determine whether the input data are erroneous. These methods are also limited to rule making. The crowdsourcing-based methods, on the other hand, collect answers from crowds at a high cost and latency, making external information-based methods less practicable for big data processing.

In the past few years, with the advances in deep learning, particularly the distributed representation of words (also known as word embeddings), researchers proposed the use of deep learning methods to deduce error data. A simple way to detect errors is separating error data from correct data via a classification model; however, due to the low error rate in real datasets, it is quite difficult to obtain a sufficient amount of error data to train a classification model. The imbalanced training data (also known as clean data and error data) will certainly lead to poor performance of a model. To deal with this problem, [2] proposed augmenting training data by generating some error data with the learned error data distribution. Because the fraction of erroneous data is so small, extracting a thorough data distribution to identify the features of the erroneous data is difficult.

Since labeling error data and designing error detection rules are costly, especially when the data volume is very big, we intend to make a deep learning model to learn the distribution of clean data. For a dataset to be checked, we intend for the error tuples to be identified by a clean-data tailored model. Our deep-learning-based approach needs to address two main technical challenges:

- Error data are very limited in the real datasets, so it is hard to extract enough negative samples to train a supervised learning model.
- Without any external information of a dataset, such as predefined rules and knowledge bases, it is hard to find inconsistent data in that dataset.

To tackle the problem of class balance in real datasets, we treat error data as outliers and train a clean-data tailored model in which parameters are only suitable for clean data. Inspired by traditional outlier detection methods, we try to learn the potential features of clean data through the distributed representation of the data. Suppose there is a model that can map the input data into a latent space and reconstruct output data by the latent representation, the model tries to minimize the distance between input and output in the training epochs. Intuitively, as long as the model performs well, it can evaluate whether the test data have the same distribution as the training data by measuring the distance between their input embedding and output embedding, because parameters in the model are only suitable for data similar to the training dataset (i.e., clean data). In this paper, we propose a framework that can recognize error data as outliers by learning the latent features of clean data.

We present SAT-GAN, a rule-free error detection model, by merging the generative adversarial network and the self-attention mechanism. Based on the Auto-Encoder Generative Adversarial Network (AEGAN) [3], SAT-GAN can capture both the attributed-level and tuple-level features of clean data and judge whether the test data have the same features and distribution with those clean data. To learn the latent distribution of data, the encoder uses a pre-trained language model to obtain the structural features, and a self-attention mechanism to capture the semantic information of data. The encoder and decoder in the SAT-GAN are trained to learn the distribution of the clean data, while the discriminator is trained to identify whether the test data satisfy that distribution. The discriminator will recognize data with different features from the clean data as error data.

### Contributions

The main contributions of our work are as follows.

- We propose an AEGAN-based deep learning model SAT-GAN to detect errors in relational datasets without any predefined rules. Associating self-attention mechanism with the pre-trained language model, our model can capture semantic features of the dataset, especially functional dependency between attributes, so that no rules or constraints are needed for our model to identify inconsistent data.
- For the lack of negative training data, we propose to train our model via zero-shot learning. As a clean-data tailored model, SAT-GAN tries to recognize error data as outliers by learning the latent features of clean data. To our best knowledge, we are the first to propose to detect errors (i.e., inconsistent data in this paper) in relational datasets by zero-shot learning.
- In experimental evaluation, SAT-GAN achieves an average $F_{1}$-score of 0.95 on five datasets, which yields at least 46.2% $F_{1}$-score improvement over traditional rule-based methods and outperforms state-of-the-art deep learning approaches in the absence of rules and negative samples.

## 2. Related Works

In this section, we briefly introduce the related works. Section 2.1 focuses on the introduction and analysis of the current advanced error detection methods. Some recent works about self-attention mechanism are given in Section 2.2. Some state of the art research for GAN is illustrated in Section 2.3.

### 2.1. Error Detection

Error detection is an important step for data cleansing. Different algorithms have been proposed to solve the problem. Available error detection methods can be divided into the following categories: rule-based methods, external information-based methods, and statistical learning-based methods [4].

Rule-based methods tend to solve the problem by detecting whether the data violate the given rules or not [5]. For instance, many of the proposed methods rely on the violation of Conditional Functional Dependencies (CFDs) [6,7] and Denial Constraints (DCs) [8] to discover inconsistent data, and rely on matching rules to identify duplicated records [9]. These algorithms rely on precise rules, which are often costly to develop.

The external information-based methods perform better than the rule-based methods for taking the knowledge bases [10] or crowdsourcing [4,11,12] as master data. The goal of knowledge base-based methods is to find a clean database with consistent data and minimal difference from current database. For example, Hao, S. et al. propose to clean relation data by building connections between a relation and a knowledge base such as Yago [13] or DBpedia [14]. The crowdsourcing-based error detection methods [4,11] try to verify the prediction results of the heuristic algorithm by the crowd. Though above methods may cost much and have high latency, they always achieve better performance than those rule-based methods by bringing human insight into tasks.

In the past few years, as deep learning has shown great promise in dealing with massive data from different domains, researchers proposed to use statistical learning methods to deduce error data. Some of the existing approaches [2] try to learn the latent relationship in the dataset and capture data error via a binary classifier. For the lack of negative samples, they tried to augment training data by generating some error data. Unlike these methods, we propose to extract features only from positive samples when facing the class imbalance problem.

### 2.2. Self-Attention Mechanism

Self-attention mechanism [15] is proposed to solve the problem that recurrent neural network cannot perform parallel computing. Currently, self-attention mechanisms are primarily used in natural language processing and computer vision. Based on work from [15], refs. [16,17] design some pre-trained language models that use the self-attention mechanism to extract high-level semantic features in sentences. All of them achieve state-of-the-art performance on several NLP tasks. In the field of computer vision, self-attention mechanisms are often used for object detection [18,19], image classification [20], and so on. To the best of our knowledge, we are the first to introduce the self-attention mechanism into an error detection model.

### 2.3. GAN

GAN is a common model for anomaly detection. One of the most influential works using a generative adversarial network comes from [21]. The authors first train a generator and discriminator using only normal images. In the next stage, they utilize the pretrained generator and discriminator by freezing the trained weights and remap the input to the latent vector. During inference, the model pinpoints an anomaly by outputting a high anomaly score, reporting significant improvement over the previous work. The main limitation of this work is its computational complexity since the model employs a two-stage approach, and remapping the latent vector is extremely expensive. Following this study, Zenati et al. [22] investigate the use of BiGAN [23] in an anomaly detection task, examining joint training to map from image space to latent space simultaneously and vice versa.

## 3. Error Detection via Zero-Shot Learning

In this section, we define the error detection problem and describe how to detect errors in relational tables with zero-shot learning.

### 3.1. Problem Statement

**Definition** **1.** *(Error entry) Given a database instance D with n attributes $A=\{attr_{1},attr_{2},\ldots ,attr_{n}\}$ and m tuples $T=\{t_{1},t_{2},\ldots ,t_{m}\}$, each tuple $t_{i}$ in D is a collection of cells $t_{i}=\left\{t_{i}\left[attr_{1}\right],t_{i}\left[attr_{2}\right],\ldots ,t_{i}\left[attr_{n}\right]\right\}$. Suppose $v^{*}$ and v are the true value and the observed value of a cell $t_{i}\left[attr_{k}\right]\left(i=1,\ldots ,m;\;k=1,\ldots ,n\right)$, respectively, $t_{i}\left[attr_{k}\right]$ is defined as an error entry of $t_{i}$ if $v^{*}\neq v$.*

Given a relational dataset *D* without any predefined rules or constraints, our objective is to detect all error entries by a clean-data tailored model.

### 3.2. Solution Overview

We formulate the error detection problem in relational tables as an outlier/anomaly detection task to settle the problem of lacking negative samples. Furthermore, we propose an AEGAN-based model called SAT-GAN that combines the advantages of generative adversarial network and self-attention mechanism to capture semantic features of the dataset, especially functional dependency between attributes, which can identify error entries in relational tables without any predefined rules or constraints by zero-shot learning.

**Figure 1:**
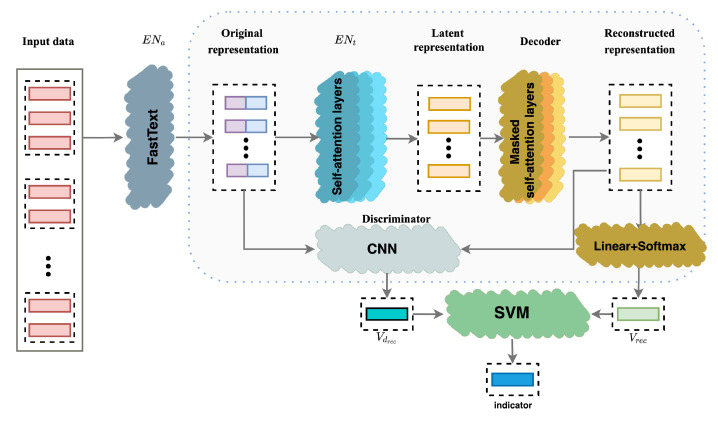
Framework overview.

Figure 1 illustrates the overview of our model, which mainly contains encoder, decoder, discriminator, and indicator generation modules. The encoder and decoder layers form an auto-encoder network, which aims to learn the cells’ representation from different levels. The decoder and discriminator compose a generative adversarial network to distinguish the clean and error data. The decoder here behaves as the generator of GAN. Since the generator module corresponds to the decoder module, we use the two terms interchangeably throughout the paper.

During data processing, each cell (i.e., entry) in *D* will be mapped into a one-hot embedding and divided into a token sequence. For a tuple $t=\{t\left[attr_{i}\right]|i\in \left\{0,1,\ldots ,n\right\}\}$ in D, our model first maps each cell $t[attr_{i}]$ in *t* into a distributed representation (denoted by $t{\left[attr_{i}\right]}_{or}$) from local level (i.e., cell level) via a pre-trained language model. Specifically, by using FastText [24], $t{\left[attr_{i}\right]}_{or}$ can capture some semantic features from both character level and word level. Naturally, *t* is represented as $t_{or}$ = concat($t{\left[attr_{1}\right]}_{or}$,$t{\left[attr_{2}\right]}_{or}$,…,$t{\left[attr_{n}\right]}_{or}$). Then, the encoder adds global level features into the representation of $t[attr_{i}]$. Instead of embedding predefined rules into the data distribution directly, we learn the implicit representation of the interaction between different attributes via the self-attention mechanism. Taking advantage of the self-attention mechanism, the encoder can learn interaction features between cells of *t* through the multi-head self-attention layer. By the encoder module, we finally obtain the latent representation of $t[attr_{i}]$ denoted by $t{\left[attr_{i}\right]}_{lr}$. Correspondingly, the latent representation of *t* is denoted by $t_{lr}$ = concat($t{\left[attr_{1}\right]}_{lr}$,$t{\left[attr_{2}\right]}_{lr}$,…,$t{\left[attr_{n}\right]}_{lr}$). The objective of the decoder is to decode the latent information and reconstruct the original representation of data. The decoder consists of several masked multi-head attention and cross-attention layers. By the self-attention layers, $t_{lr}$ will be converted into the reconstructed representation $t_{rr}=concat\left(t{\left[attr_{1}\right]}_{rr},t{\left[attr_{2}\right]}_{rr},\ldots ,t{\left[attr_{n}\right]}_{rr}\right)$. After that, $t{\left[attr_{i}\right]}_{rr}$ is mapped to a probability vector $t{\left[attr_{i}\right]}_{rs}$ through the linear and softmax layer, in which each item corresponds to a candidate reconstructed cell.

Finally, the discriminator is used to distinguish the features between the clean and error data. Though only clean data are fed into the model, the discriminator tries to measure the reconstruction distance (denoted by $d_{attr_{i},or}$) between $t{\left[attr_{i}\right]}_{or}$ and $t{\left[attr_{i}\right]}_{rr}$ for $A_{i}$. Concatenating all $d_{attr_{i},or}$ of *t* and normalizing these data, the model outputs an vector $V_{d_{rec}}$, whose items are non-zero constants.

To generate the indicator vector, an SVM model, followed by the discriminator, is trained to learn the threshold for distinguishing clean entries and error entries. Comparing $t{\left[attr_{i}\right]}_{rs}$ with the original one-hot embedding of cells, we can know whether the cell is correctly reconstructed or not.

### 3.3. Encoding Data Distribution from Multiple Levels

As the first module, the encoder learns the data distribution from the input and converts raw data into the latent representation containing computational semantics. For embedding the input data, encoder tries to capture statistical features of cells from local and global level, respectively. Local level (i.e., attribute level) features reflecting the distribution of values and formats are captured via the $EN_{a}$ module, while global level (i.e., tuple level) features reflecting the joint distribution of different attribute values are captured via $EN_{t}$ module.

In $EN_{a}$, FastText is used to initialize the original embeddings of cells from the local level. The embeddings are taken at a character and word level tokens [25]. For any $t[attr_{i}]$ in a tuple *t*, the cell level embedding layer converts it into a vector [$t_{o1}^{attr_{i}},t_{o2}^{attr_{i}}$], where $t_{o1}^{attr_{i}}$ and $t_{o2}^{attr_{i}}$ represent the character and word embeddings of $t[attr_{i}]$, respectively. Concatenating all cells’ representation, we can obtain the distributed representation of *t*, i.e., [$t_{o1}^{attr_{1}},t_{o2}^{attr_{1}}$,$t_{o1}^{attr_{2}},t_{o2}^{attr_{2}}$, …, $t_{o1}^{attr_{n}},t_{o2}^{attr_{n}}$].

**Figure 2:**
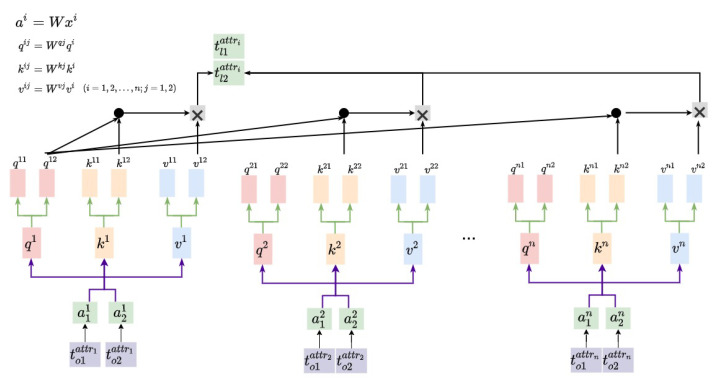
Details of the multi-head self attention in $EN_{t}$.

Following the cells’ embedding module, the tuple embedding module $EN_{t}$ employs a multi-head Self-Attention Mechanism (MSA) to characterize global features of the tuples. Since the $EN_{a}$ extracts two-level features of cells, we set the number of heads in the MSA to two. The details of $EN_{t}$ are shown in Figure 2. It reads the output of $EN_{a}$, and forwards each of the cell level embedding $t_{o_{j}}^{attr_{i}}$(i=1,2,…,n;j=1,2) to three full connection (FC) layers separately. These FC layers perform matrix transformation, and output three different feature embeddings of $\left[t_{o1}^{attr_{i}},t_{o2}^{attr_{i}}\right]$, which are denoted as $\left[q^{i1},q^{i2}\right]$, $\left[k^{i1},k^{i2}\right]$, $\left[v^{i1},v^{i2}\right]$. Parameter matrices in FC layers satisfy $W^{qj}\in R^{d_{model/2},d_{q}}$,$W^{kj}\in R^{d_{model/2},d_{k}}$,$W^{vj}\in R^{d_{model/2},d_{v}}$, and we set $d_{model}=100$, $d_{q}=d_{k}=d_{v}=d_{model}/2=50$. Then, with the adoption of the multi-head attention, $EN_{t}$ calculates the interaction SA between different attributes via the following formulas.

(1) $$\begin{array}{c} {SA}_{h}=softmax\left(\frac{q_{h}K}{\sqrt{d_{k}}}\right) \end{array}$$

(2) $$\begin{array}{c} SA=concat\left(SA_{1},SA_{2}\ldots ,SA_{h}\right) \end{array}$$

In Equation (1), *K* denotes an *n*-columned matrix where each row $k_{i}$ is the output of FC layer, and $d^{k}$ is the dimension of $t[attr_{i}]$. Furthermore, the tuple level representation of $t[attr_{i}]$ can be denoted by SA·V where V=K.

After that, $EN_{t}$ concatenates the tuple level representation of each cell $t^{attr_{i}}$, and outputs the latent data representation of *t* denoted by [$t_{l1}^{attr_{1}},t_{l2}^{attr_{1}}$,$t_{l1}^{attr_{2}},t_{l2}^{attr_{2}}$, …, $t_{l1}^{attr_{n}},t_{l2}^{attr_{n}}$].

### 3.4. Reconstructing Original Distribution by the Decoder

The basic objects of the auto-encoder in our model are mapping the input data to a latent space and reconstructing the original representation. Due to the need for a self-attention mechanism in the encoder module, we prefer to choose transformer [15] as the base auto-encoder of our model.

For the generator layer G in the generative adversarial network, we try to reconstruct the original distribution of input data via the decoder of the transformer. To reduce the parameters of the model, the visibility matrix [26] is used in the decoder module as an attention mask so that each token can only aggregate information from other structurally related tokens during the self-attention calculation.

**Definition** **2.** *(visibility matrix) Given two tokens p and q, if p and q are in the same cell (or entry), we say that they are visible to each other. For a cell $t[attr_{i}]$, we say $t[attr_{j}]$ is visible to $t[attr_{i}]$ when at least one token in $t[attr_{j}]$ is visible to any token in $t[attr_{i}]$. The visibility matrix M is a symmetric binary matrix with $M_{i,j}=1$ when $t[attr_{j}]$ is visible to $t[attr_{i}]$, and $M_{i,j}=0$ otherwise.*

**Figure 3:**
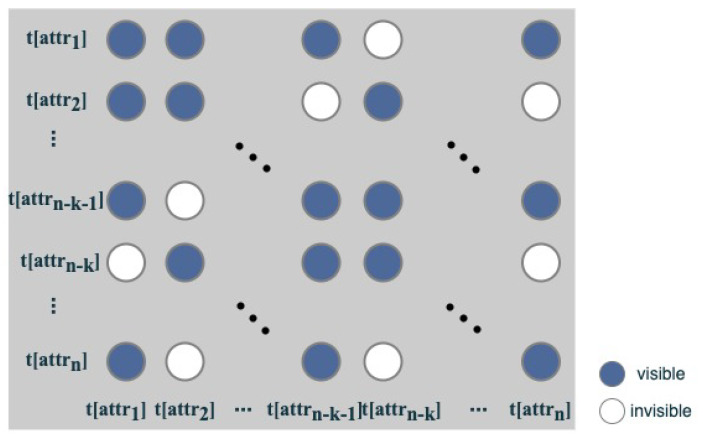
Graphical illustration of visibility matrix.

Specifically, tokens here can be characters in a cell. Figure 3 graphically illustrates the visibility matrix.

In the decoder layer, we calculate the masked multi-head self attention as follows:

(3) $$\begin{array}{c} {SA}_{h}=softmax\left(\frac{q_{h}K}{\sqrt{d_{k}}}\right)M_{h} \end{array}$$

(4) $$\begin{array}{c} SA=concat\left(SA_{1},SA_{2}\ldots ,SA_{h}\right) \end{array}$$

In Equation (3), different heads employ different visibility matrices during the multi-head attention calculations.

For the head that aggregates information at the character level, we set $M_{ij}$ = 0 if $t[attr_{j}]$ is not visible to $t[attr_{i}]$. The word-level information is sparse compared to the character-level information, so we set M=1 for the head aggregating information at the word level. Reconstructed representations from the decoder are denoted as $t_{rr}=\left[t_{r1}^{attr_{1}},t_{r2}^{attr_{1}},t_{r1}^{attr_{2}},t_{r2}^{attr_{2}},\ldots ,t_{r1}^{attr_{n}},t_{r2}^{attr_{n}}\right]$.

### 3.5. Discriminating Error Data

Since there are no negative samples to train the model with supervised learning, we attempt to train the model via the adversarial training so that it can extract the hidden features of the data well.

The discriminator module is used to determine whether the input data are clean, which is a Convolutional Neural Network (CNN) introduced in TextCNN [27]. It takes the original data representation and reconstructed data representation as input, and identifies whether the input representation is from the clean data.

We co-train the encoder, generator, and discriminator via adversarial training. During training, we define two kinds of losses:

- Adversarial Loss. To reduce the instability of GAN training, we define the feature matching loss for adversarial learning as follows.
  (5) $$L_{adv}=E_{x∼p_{X}}∥f\left(x\right)-E_{x∼p_{X}}f\left({G\left(x\right)∥}_{2}\right.$$
  where G(x) is the reconstructed data, and f is a function that outputs the intermediate features that are extracted from the discriminator.
- Reconstructed Loss. We employ the reconstructed loss to penalize the generator by measuring the distance between the original and reconstructed representation (z=G(x)).
  (6) $$L_{rec}=E_{x∼p_{X}}{∥G(x)-x∥}_{1}$$

Overall, our objective function for the generator is as follows:

(7) $$L=w_{adv}L_{adv}+w_{rec}L_{rec}$$

### 3.6. Generating Error Indicator

We infer error entries via a distance vector $V_{d_{rec}}$, which can be obtained from the discriminator. The indicator is an *n*-dimensional vector (*n* is the number of attributes in a tuple). Items in the indicator are real numbers in [0, 1].

In Section 3.5, we construct a CNN network to judge whether the input data are clean or not. It takes the original and reconstructed data representation as input, and use L1 regularization as distance between two representations (called reconstructed distance). During the phase of inference, we take an SVM model and collect these distances as training data, so that the SVM model is trained to learn the distribution of clean data’s reconstructed distance. When inputting the reconstructed distance of a tuple into the SVM model, data anomalies in any dimension will be labeled as 1, which means the corresponding attribute value may be erroneous.

### 3.7. Running Example

**Figure 4:**
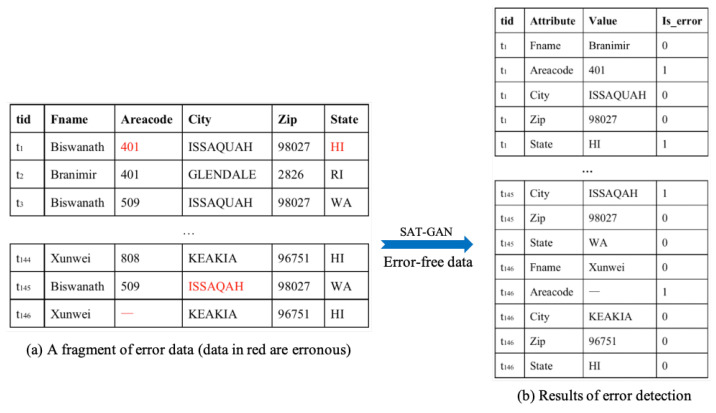
Running example.

**Figure 5:**
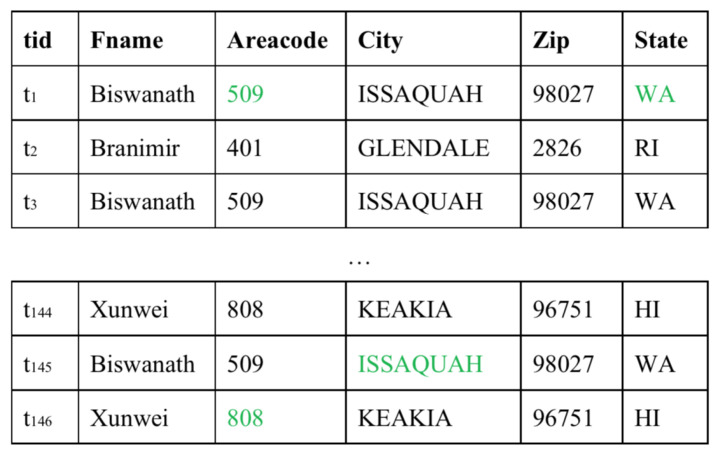
An fragment of error-free data. Data in green are the ground truth for the error data shown in Figure 4a.

In this section, we illustrate how the model detects dirty data, using a dirty data fragment with the five attributes shown in Figure 4a as an example. Figure 5 gives the corresponding ground truth of data in Figure 4a.

During data preprocessing, we embed all data in the dataset with one-hot embedding. In the training phase, SAT-GAN is fed with a set of clean data, thus learning only the features of the clean data and acquiring parameters that apply only to the clean data. In the detection phase, we feed tuples wit errors into the model in turn. Taking $t_{1}$ as an example. First we feed $t_{1}=\left[Biswanath,401,ISSAQUAH,98027,HI\right]$ into the model and obtain the original representation $t_{or}^{1}$, and the encoder encodes $t_{1}$ to $t_{lr}^{1}$. Then the decoder in SAT-GAN with masked self-attention layers decodes $t_{lr}^{1}$ into $t_{rr}^{1}$, both $t_{or}^{1}$ and $t_{rr}^{1}$ are represented by attribute level embedding and tuple level embedding.

The discriminator receives $t_{or}^{1}$ and $t_{rr}^{1}$, and calculates the distance between them which is denoted by $V_{d_{rec}}=\left[d_{t_{1}}^{Fname},d_{t_{1}}^{Areacode},d_{t_{1}}^{City},d_{t_{1}}^{Zip},d_{t_{1}}^{State}\right]$. In this example, $V_{d_{rec}}$ is a five-dimensional vector with each dimensional data representing the reconstructed distance on the corresponding attribute. Using the SVM, the model transforms $V_{d_{rec}}$ into a vector of error indications, with each item in the vector being either 0 or 1. As shown in Figure 4b, all erroneous entries such as $t_{1}.Areacode$ = “401” and $t_{1}.State$ = “HI” in $t_{1}$ are marked as 1 and clean entries are marked as 0.

## 4. Experiments

In this section, we compare our method with existing error detection methods. We seek to validate the effectiveness of our model and whether self-attention is the important mechanism for our model to capture relationship between attributes.

### 4.1. Experimental Environment

The SAT-GAN was implemented by PyTorch on a computer with a single NVIDIA GeForce RTX 3090 GPU (24 GB Memory) with Inter(R) Xeon(R) Gold 6226 R CPU @ 2.9 GHz and 128 GB memory.

### 4.2. Experimental Setup

**Datasets.** We experimented on five datasets from different fields to evaluate the performance of the proposed model on error detection. Table 1 summarizes information on these datasets. The error types are Missing Value (MV), Typo (T), Formatting Issue (FI), and Violated Attribute Dependency (VAD) [28]. As shown in the table, the datasets span different sizes and have different type of errors:

- The Hospital dataset is a real-world dataset based on health care providers and hospitals, which has 100k tuples and 19 attributes. This dataset is used in several data cleaning papers [2,29].
- The Flights [30] contains data on the departure and arrival time of flights from different data sources. It contains missing values, formatting issues, and values that violated attribute dependency. The error rate of this dataset is about 30%. Ground truth information is available for all cells.
- The Movies is a dataset from the Magellan repository [31] with an error rate of 6%. Errors in this dataset include missing values and formatting issues.
- The Soccer dataset provides information about football players and their teams. Adult is a set of census data collected from the UCI repository. Both Soccer and Adult are provided by Rammerlaere and Geerts [7]. In order to simulate real data, we set the error rate of test dataset to 3%. Error entries were introduced artificially with BART [32] by injecting typos and swapping cell values.

We divided our experimental study into two parts: performance evaluation and ablation study. The performance evaluation part was mainly used to test the effectiveness of the whole model, while the ablation experiment was mainly used to verify the importance of self-attention for model performance improvement.

**Evaluation Metrics**. We evaluate our method on three metrics:

(1) Precision (P): The number of correctly detected cells over the total number of detected cells;
(2) Recall (R): The number of correctly detected cells over the total number of dirty cells;
(3) F1-score: F1-score is computed as 2(P×R)/(P+R).

For our model, we used the pre-trained FastText 50-dimensional word embedding [25] and fixed the embeddings during training. For model learning, we used 60/20/20% train/dev/test split; we used the Adam algorithm as optimization for our model and train models for 50 epochs with a batch-size of 128.

**Baselines.** We compare our approach with the following alternatives.

- Outlier Detection (OD): This method follows a correlation-based outlier detection approach. Given a cell corresponding to an attribute $A_{i}$, the method considers all correlated attributes in $A\backslash A_{i}$ with it to detect an outlier based on the pair-wise conditional distributions [33,34].
- Constraint Violations (CV): This method identifies errors by leveraging violations of denial constraints. It is a proxy for rule-based error detection methods [29].
- Forbidden Item Sets (FIS): This method captures unlikely value co-occurrences in noisy data [35]. It leverages the lift measure from association rule mining to judge how probably a value co-occurrence is, and uses this measure to identify erroneous cell values.
- Logistic Regression (LR): This method corresponds to a supervised logistic regression model that classifies erroneous or correct data. The features of this model correspond to pairwise co-occurrence statistics of attribute values and constraint violations [36].
- HoloDetect (AUG): A state-of-the-art statistic learning-based error detection method [2]. This method proposes a data representation model to capture the syntactic and semantic features of errors. It formulates the error detection problem as a binary classification task and deals with class imbalance problem by augmenting negative training datasets.
- RAHA: RAHA is a configuration-free error detection system [12] that designs specific detection algorithms for certain errors. It classifies the data errors as semantics errors and syntactic errors, and extracts the corresponding features for each type of error to design algorithms that can detect comprehensive errors.

### 4.3. Performance Evaluation

We conducted two sets of experiments to verify our model on semantic errors and syntactic errors, respectively. The performance of our model SAT-GAN and baselines are reported in Table 2 and Table 3.

From Table 2, we can obtain following observations for semantic error detection. Our method outperforms all non-deep-learning-based methods and achieves comparable performance to the state-of-the-art approach HoloDetect. Compared with HoloDetect, neither predefined rules nor negative samples are needed for our method. By combining the generative adversarial network with the self-attention mechanism, our model can effectively detect errors in relational tables. Specifically, our model significantly outperforms those non-deep learning based methods (i.e., OD, CV, BIS, and LR) in the absence of predefined rules or constraints, and achieves at least 46.2% F1-score improvements correspondingly on different datasets. Even compared with the state-of-the-art deep learning model AUG, our method achieves F1-score improvements on Hospital and Soccer datasets, and has comparable performance on Adult dataset. Furthermore, the average F1-score of our model (0.969) over three experimental datasets outperforms that of AUG. Different from AUG, our model only embeds the attribute level features in the data distribution part and does not need negative training data. The comparable performance is mainly from self-attention mechanism, which can capture dependency relationship between attributes. In fact, these relationships may potentially represent the tuple level features. Without error data, our model also performs well (with F1-score all above 0.955) mainly due to the fact that the adversarial network has learned the characteristics of clean data and it is hard for erroneous data to fool this network.

From Table 3, we can see that our model performs better than RAHA in precision and F-measure when dealing with syntactic errors, but is slightly inferior to RAHA on recall. By classifying the data errors, RAHA extracts the corresponding features for each type of errors and designs corresponding algorithms for each specific error types, which improve its recall.

Overall, our method is quite robust on datasets from different fields and different errors. Specifically, our model achieves both high precision and F1-score in most cases. In this paper, we formulate the tuple level error checking problem as outlier detection task. Furthermore, due to the adversarial network for accurately finding abnormal distributed data, our model achieves high precision. In addition, our algorithm performs well for data sets with relatively high error rates (i.e., Fights).

### 4.4. Ablation Study

In this section, we perform ablation studies to evaluate the importance of self-attention mechanism for our model SAT-GAN. We replace the self-attention mechanism in the auto-encoder part with DNN, which does not specifically learn the interaction features between cells of a tuple. The alternative model with DNN as the auto-encoder is denoted by vanilla-AEGAN. Table 4 shows the performance comparison of two models.

From Table 4, we can see that SAT-GAN achieves obviously higher performance than vanilla-AEGAN both on precision and recall. With the self-attention mechanism, the F1-score improvements by SAT-GAN are significant with the growth rates of 31.9%, 11.8%, and 12.9% on Hospital, Soccer, and Adult datasets, respectively, which is positively correlated with attribute numbers of the datasets. Especially, Hospital has the most number of attributes among the three datasets, and the F1-score improvement by SAT-GAN on this dataset is the greatest. In contrast, with the least number of attributes among the experimental datasets, the F1-score improvement on Soccer is the smallest correspondingly. The experimental results clearly demonstrate that self-attention mechanism in our SAT-GAN model are effective for error detection by extracting semantically relevant features between attributes. In fact, the more attributes in a dataset, the more semantically relevant features can be captured by self-attention mechanism in SAT-GAN. Furthermore, the vanilla-AEGAN still achieves better F1-score than those non-deep learning methods, validating the effectiveness of our error detection method based on clean data.

## 5. Conclusions and Future Works

We propose a zero-shot learning method to detect errors in relation tables, which constructs an AEGAN-based model SAT-GAN to capture the global features. SAT-GAN can judge whether an input record has the same features and distribution with clean data or not on the global level and detects error cells through attention maps on the local level. In contrast to those methods that perform data augmentation to solve the class imbalance problem, our method learns only the features of clean data, thus avoiding the problem that the learned distribution features deviate from the true distribution due to inadequate sampling of negative samples. Experimental results on real-world datasets show that our model outperforms existing rule-based methods, and achieves comparable performance to state-of-the-art supervised approaches in the absence of rules and negative training data; however, our approach performs poorly on particularly similar and semantically sound data. In future work, we will improve the distributed representation of data to obtain better performance.

## Figures and Tables

**Table 1:** Dataset statistics.

Datasets	Size	Attributes	Error Types
Hospital	100 k	19	T
Flights	2 k	7	MV,FI,VAD
Movies	7 k	17	MV,FI
Soccer	200 k	10	T,VAD
Adult	97 k	11	T,VAD

**Table 2:** Performance of semantic error detection.

Method	Hospital			Soccer			Adult		
	Precision	Recall	F1-Score	Precision	Recall	F1-Score	Precision	Recall	F1-Score
OD	0.640	0.667	0.653	0.999	0.051	0.097	0.999	0.001	0.002
CV	0.030	0.372	0.055	0.039	0.846	0.074	0.497	0.998	0.644
FIS	0.008	0.001	0.003	-	-	-	0.990	0.254	0.405
LR	-	-	-	0.721	0.084	0.152	0.051	0.072	0.059
AUG	0.903	**0.989**	0.944	0.922	**1**	0.959	0.994	**0.987**	**0.991**
SAT-GAN	**0.926**	0.987	**0.955**	**0.935**	0.998	**0.965**	**0.997**	0.979	0.988

**Table 3:** Performance of syntactic error detection.

Method	Flights			Movies		
	Precision	Recall	F1-Score	Precision	Recall	F1-Score
RAHA	0.85	**0.93**	0.89	0.90	**0.97**	0.93
SAT-GAN	**0.91**	0.88	**0.90**	**0.97**	0.92	**0.95**

**Table 4:** Experimental results on ablation study.

Method	Hospital			Soccer			Adult		
	Precision	Recall	F1-Score	Precision	Recall	F1-Score	Precision	Recall	F1-Score
SAT-GAN	0.926	0.987	0.955	0.935	0.998	0.965	0.997	0.979	0.988
vanilla-AEGAN	0.813	0.652	0.724	0.906	0.824	0.863	0.972	0.795	0.875

## Data Availability

Not applicable.
